# The Effect of Preoperative Anemia on Blood Transfusion Outcomes in Major Head and Neck Cancer Surgery

**DOI:** 10.3390/cancers17132136

**Published:** 2025-06-25

**Authors:** Munib Ali, Steven C. Nakoneshny, Joseph C. Dort, Khara M. Sauro, Thomas Wayne Matthews, Shamir P. Chandarana, Todd A. Wilson, David C. McKenzie, Christiaan Schrag, Jennifer Matthews, Robert D. Hart

**Affiliations:** 1 Department of Family Medicine, Cumming School of Medicine, University of Calgary, Calgary, AB T2N 1N4, Canada; munib.ali@ucalgary.ca; 2 Ohlson Research Initiative, Arnie Charbonneau Cancer Institute, Cumming School of Medicine, University of Calgary, Calgary, AB T2N 1N4, Canadajdort@ucalgary.ca (J.C.D.);; 3 Department of Community Health Sciences, Cumming School of Medicine, University of Calgary, Calgary, AB T2N 1N4, Canada; 4 Section of Otolaryngology-Head and Neck Surgery, Department of Surgery, Cumming School of Medicine, University of Calgary, Calgary, AB T2N 1N4, Canada; 5 Section of Plastic Surgery, Department of Surgery, Cumming School of Medicine, University of Calgary, Calgary, AB T2N 1N4, Canada

**Keywords:** head and neck, microvascular reconstruction, evidence-based research, epidemiological studies, anemia, intravenous iron, surgical oncology, blood transfusion, predictive modeling, enhanced recovery after surgery (ERAS)

## Abstract

**Background/objectives**: Major head and neck oncologic surgeries requiring microvascular reconstruction frequently result in complications such as perioperative blood transfusion (PBT). Not only are blood products overutilized and associated with risks, but preoperative anemia is both a modifiable and predisposing factor for PBT. Our objective was to assess risk factors for PBT and determine a high-risk preoperative hemoglobin to inform transfusion stewardship practices. **Methods**: Patients that underwent head and neck cancer free flap reconstruction (*n* = 363) between 2012 and 2019 were included. Univariable and multivariable analyses evaluated predictors of PBT. **Results**: Overall, 11% of patients were anemic and 19% were transfused. Mean preoperative hemoglobin was significantly lower in the PBT group (128 g/L vs. 145 g/L, *p* < 0.0001). In our multivariable model, lower preoperative hemoglobin (odds ratio [OR] = 0.94), higher T stage (OR = 2.65), and lower body mass index (BMI) (OR = 0.89) increased the odds of PBT. Adjusting for staging and BMI, the OR of PBT was increased below 120 g/L hemoglobin. Higher mean units of PBT were administered for hemoglobin below 150 g/L with a large inflection below 120 g/L (*p* < 0.0001). **Conclusions**: Low preoperative hemoglobin is the strongest predictor of PBT in major head and neck cancer surgery. Recognizing and managing anemia is essential in surgical planning.

## Simple Summary

Perioperative blood transfusion (PBT) is a common complication following free flap surgery. Given the risks, costs, and overuse of blood products, this investigation aimed to estimate a high-risk preoperative hemoglobin threshold for PBT in patients undergoing major head and neck oncologic surgery. Based on our findings, preoperative hemoglobin was not only the strongest predictor of PBT usage, but there was a large increase in the average number of units of blood products utilized below the threshold of 120 g/L. Anticipating, preventing, and treating anemia prioritizes patient health outcomes and transfusion stewardship. Emerging preoperative therapies such as intravenous iron therapy show promise with respect to reducing unnecessary PBT.

## 1. Introduction

Advancements in the surgical reconstruction of the head and neck have had remarkable impacts on the survival and quality of life for patients with head and neck cancer (HNC) [1,2]. In major HNC surgery, free flaps are often required to repair structures vital for breathing, speaking, and swallowing. The complex anatomy, extensive surgical resections, and overall length of these procedures can predispose patients to perioperative blood transfusion (PBT).

Red blood cell transfusion is a homologous tissue transplant that carries with it the proinflammatory apparatus of the donor. The resulting immunomodulation can lead to negative outcomes, particularly in patients with cancer, such as tumor recurrence and lower overall survival [3,4,5,6,7]. These findings have been replicated in many other sites of malignancy [8,9,10]. Along with immune dysregulation, PBT is costly and can increase the risk of transfusion-related acute lung injury, transfusion-associated circulatory overload, and hemolytic or allergic reactions [11]. Therefore, it is important in the management of surgical patients to avoid inappropriate overuse of PBT and reduce the usage of blood products where possible.

Surgeons must maintain a balance between maintaining optimal systemic and flap hemodynamics and the potential sequelae of a transfusion. A routinely collected, modifiable risk factor that predisposes patients to PBT is low preoperative hemoglobin (Hgb) or preoperative anemia [12,13]. HNC patients often present with low preoperative Hgb secondary to nutritional deficiencies and anemia of chronic disease [14]. The World Health Organization (WHO) currently defines anemia as a blood Hgb concentration below 120 g/L in adult, non-pregnant females and below 130 g/L in adult males [15]. The presumed reason for most PBT’s is intraoperative blood loss in addition to the pre-existing anemia. Szakmany and colleagues (2006) observed that the degree of preoperative anemia, which can increase the need for PBT in perioperative management, reduces disease-specific survival in HNC surgery, likely owing to the dose-dependency of immune dysregulation that increases with the volume of blood transfused [16]. Moreover, anemia is independently associated with poorer disease prognosis, postoperative complications, morbidity, and mortality following HNC surgery [17,18,19]. Many patients undergoing major HNC surgery require adjuvant radiation and/or chemotherapy and perioperative anemia can also portend a worse prognosis in these cases [20]. Of note, PBT does not improve these poorer outcomes seen with low Hgb [20]. Since the postoperative recovery following free flap surgery is physiologically demanding, anemia is anticipated to worsen over the course of hospitalization.

Given that many HNC patients present with low Hgb or anemia in preoperative consultation, this provides a practical opportunity for potential preoperative therapy. However, little is known about the critical threshold at which intervention may benefit these patients with low preoperative Hgb. The purpose of this study was to understand factors associated with PBT and estimate a high-risk preoperative Hgb threshold for PBT to inform clinical practice. Implications for preoperative therapy alongside current transfusion practices are discussed.

## 2. Materials and Methods

### 2.1. Study Design and Setting

This prospective cohort study was conducted in Calgary, Alberta, Canada, between June 2012 and June 2019. Patients included in the cohort were seen at a tertiary academic head and neck oncology program.

### 2.2. Patient Selection

The prospectively collected data were retrospectively analyzed for consecutive adult patients (age > 18 years) undergoing major head and neck surgery with free flap reconstruction. Flap cases for osteoradionecrosis and benign pathology were excluded. The Strengthening the Reporting of Observational Studies in Epidemiology (STROBE) checklist was utilized to ensure transparent reporting.

### 2.3. Variables

The exposure variable was preoperative HGb, measured in g/L, recorded prospectively. Preoperative anemia was defined as Hgb < 120 g/L (dichotomous variable, anemic or not). Only data on packed red blood cells was collected. Data on other blood products such as fresh frozen plasma or platelets were not included.

The outcome variable was PBT (dichotomous, transfusion or not), which was abstracted from the Alberta Health Services Patient Blood Management Program. To obtain PBT data, eligible patients were deterministically linked to the blood management program using their medical record number and their transfusion data.

Baseline patient demographic characteristics included age (continuous), sex (dichotomous), comorbidities (diabetes, Chronic Obstructive Pulmonary Disease (COPD), hypertension, and heart disease, each dichotomous), stage at diagnosis (T, N, and overall, categorical), history of smoking and alcohol use (categorical), body mass index (BMI, continuous and dichotomized), cancer recurrence (dichotomous), primary cancer site (categorical), and type of cancer (categorical). Intraoperative variables included type of flap (radial forearm, fibular, anterolateral thigh) and complications (Clavien-Dindo).

We visually inspected the data to explore missingness. There were no missing data for the outcome variable (PBT) but data were missing for 8.0% (*n* = 30) of the cohort for the exposure variable (pre-operative HGb). When we compared those with missing exposure data to those without missing exposure data, there were no differences (Table S1). With regards to missingness in covariate data, there was missing data on cancer stage for 19.8% of patients, but when considering those who did not have a neck dissection (no N stage), there were only 1.4% of patients who had missing cancer stage data. Similarly, there were 8% of patients with no BMI, which seemed to be missing at random when comparing those with and without missing BMI data.

### 2.4. Statistical Analyses

All statistical analyses were performed using Stata 17.0 [21]. Categorical variables were compared using a chi-square or Fisher’s exact test. Continuous variables were compared using appropriate parametric or non-parametric analyses. A *p* value < 0.05 was considered significant. Clinically relevant variables of interest that were significant using the comparisons were compared using a univariate logistic regression. Univariate regression results of *p* < 0.05 were included in a multivariable logistic regression model. The final model was derived using backwards stepwise logistic regression. The odds ratios for PBT were adjusted for clinically relevant variables found to be statistically significant in the final model. In this model, preoperative Hgb and body mass index were inputted as continuous variables, while tumor stage, node stage, Clavien-Dindo classification, and sex were categorical. The adjusted odds of PBT were also reported for every 10 g/L increment in preoperative Hgb. Mean units of blood transfused were compared at preoperative Hgb increments of 10 g/L and a secondary analysis examined transfusion utilization by year.

### 2.5. Ethical Approval

The authors used A Project Ethics Community Consensus Initiative (ARECCI) framework to assess for and mitigate ethical risks, including the ARECCI Ethics Screening Tool and the ARECCI Ethics Guidelines. The project was deemed a quality improvement initiative with minimal risk (ARECCI score = 2) [22] and as such was exempt from approval from our institutional research ethics board.

## 3. Results

### Patient Characteristics

A total of 363 HNC free flaps were included among 344 unique patients. Patient demographic data (including both preoperative variables and intraoperative variables) as well as PBT usage data are presented in Table 1. Briefly, the sample was predominately male (71%), without comorbidities (41%), and largely consisting of squamous cell carcinoma (SCC). Fifty-two percent of the cancers were late stage (Stage III or IV). The most common site of the primary tumor was the oral cavity (65%), and the most frequent flap was the radial forearm free flap (RFFF) (51%). Procedures were most often accompanied with unilateral neck dissection (54%). Major complications (or Clavien-Dindo class IIIb–V) were present in 16% of the cohort. Only 35% of patients were smokers, while alcohol consumption was more common in 64% of the sample. The average BMI was 25.7 ± 0.81 kg/m^2^, with most patients being normal in weight or overweight (74%). There were differences between those with and without PBT based on several demographic characteristics (sex, BMI, and T stage) and intraoperative characteristics (type of flap and complications) (Table 1).

Overall, 19% of the sample received PBT and 11% of patients with recorded preoperative Hgb were anemic prior to surgery. The odds (unadjusted) of receiving PBT was 0.94 (95% CI: 0.922–0.964, *p* < 0.0001) for those who were anemic compared to those who were not anemic. Using univariable analysis, we found that females with low preoperative Hgb (g/L), higher tumor staging, late-stage cancers (T3 or T4), and who were underweight (BMI < 18.5 kg/m^2^), with a higher Clavien-Dindo class (IIIb–V) and a flap type other than a radial free forearm flap, were associated with receiving PBT.

Table 2 shows the results of a multivariable analysis. Preoperative Hgb remained the strongest independent predictor of PBT (OR = 0.94, 95% CI = 0.92–0.96, *p* < 0.0001). As such, a single point increase in Hgb reduced the odds of PBT by 6%. However, alongside preoperative Hgb, late-stage cancers (2.65, 95% CI = 1.31–5.39, *p* = 0.007) and an underweight BMI (OR = 0.89, 95% CI = 0.82–0.96, *p* = 0.003) also increased the risk of requiring PBT. Having a major complication (Clavien-Dindo class IIIb–V) was excluded as it demonstrated collinearity during the backwards regression. While flap type was associated with PBT, it was excluded as it was not clinically relevant (all flap types except for the radial forearm free flap were predictive of PBT) and was highly variable.

**Figure 1:**
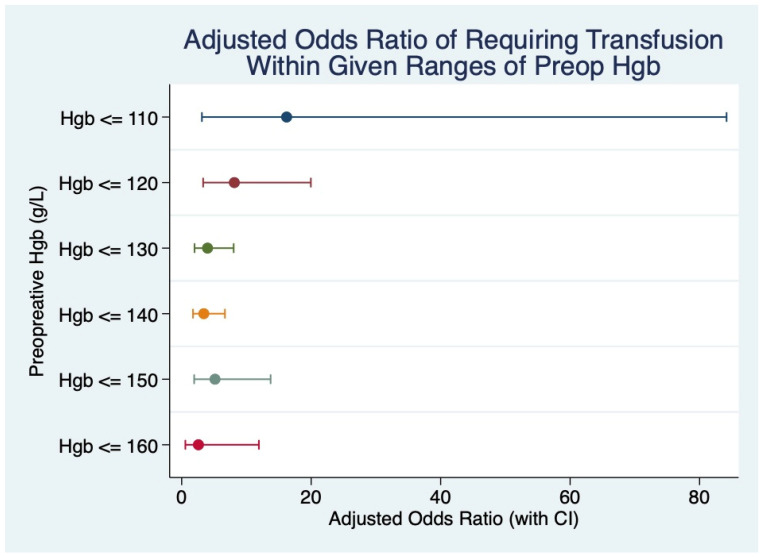
The adjusted odds (adjusted for body mass index and tumor stage) of receiving perioperative blood transfusion below discrete preoperative hemoglobin increments.

We analyzed the role of preoperative Hgb on PBT requirements in 10 g/L increments with respect to the odds ratio after adjusting for tumor stage and BMI. While the odds of receiving PBT trended higher with lower preoperative Hgb with an inflection below 120 g/L, there was variability in the data (Figure 1). Notwithstanding this variability, the adjusted odds of PBT at preoperative Hgb < 120 g/L was significant.

**Figure 2:**
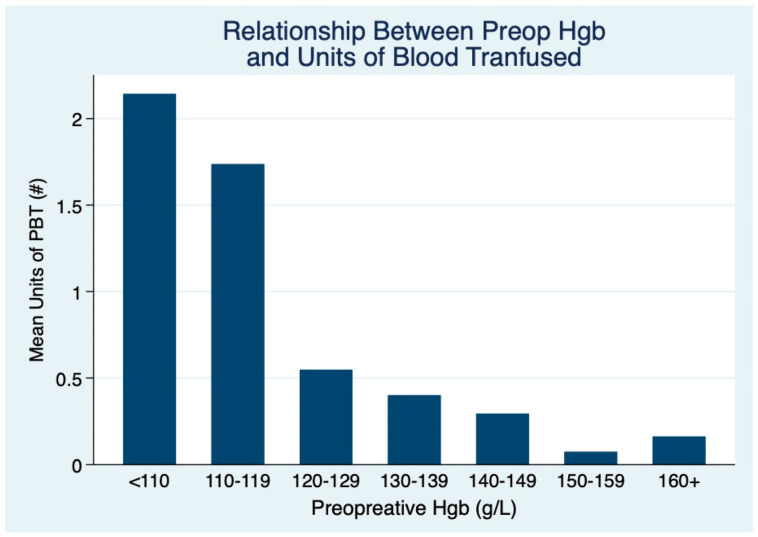
Mean units of blood transfused as a function of preoperative hemoglobin at various increments.

Mean units of blood transfused was also investigated as a function of preoperative Hgb in Figure 2. We found that a preoperative Hgb under 150 g/L can increase the mean units of blood transfused compared to those with a preoperative Hgb above 150 g/L (*p* = 0.001). However, there is additionally an upward inflection point in blood product requirement below 120 g/L Hgb. A mean 1.89 units (95% CI = 0.91–2.86) of PBT were administered for those below 120 g/L, whereas 0.27 units (95% CI = 0.18–0.35) was the average for those with a preoperative Hgb above 120 g/L. The difference in mean units of PBT remained significant up to the comparison of patients below 150 g/L preoperative Hgb at 0.59 mean units of PBT (95% CI = 0.40–0.79) and those with preoperative Hgb higher than 150 g/L at 0.11 mean units of PBT (95% CI = 0.02–0.21). This difference represents up to a relative five-fold decrease in blood product requirements if preoperative Hgb is above 150 g/L. There was no difference in transfusion utilization by year (*p* = 0.230).

## 4. Discussion

For patients with head and neck cancer undergoing free flap surgery, low preoperative Hgb, being underweight, and having a high T stage all contribute to the incidence of PBT. However, preoperative Hgb was the strongest predictor, with an increased adjusted odds of PBT below 120 g/L. We also found that the transfused group had a significantly lower preoperative Hgb, and the volume of blood required was elevated sevenfold for anemic patients (<120 g/L). Nearly one fifth (19%) of our patients went on to be transfused during their perioperative stay. Compared to other reports in major HNC surgery, a relatively low percentage of patients received PBT [23]. Furthermore, we found that 11% of our patients entered surgery with baseline anemia (Hgb < 120 g/L), which was also slightly lower than expected in HNC patients [14].

Due to the complexity of major HNC surgery, a crucial element of informed consent prior to proceeding with the operation is weighing its potential risks and complications against the desired outcome. The utilization of blood and its components can be lifesaving and clinically necessary. Although PBT may be indispensable in such scenarios, especially given that the rate of transmission of infectious disease via transfusion has become increasingly rare, some literature has associated the administration of PBT with longer hospital stay, risk of wound infections, cancer recurrence, and mortality [3,4,5,6,24,25]. Conversely, other authors have contended that PBT is not associated with worse prognosis in HNC [26,27]. Preoperative anemia, however, has been consistently recognized as an adverse prognostic indicator for HNC [19,28,29]. While the evidence for PBT as a causative factor in recurrence or disease-specific survival remains a controversial issue, there is strong evidence to suggest that it is not the solution for the problems associated with preoperative anemia [30,31].

Although there is an overall trend towards reducing transfusion in medicine, our service has not seen a significant decrease in PBT over the years. Much of the reflexive desire to initiate PBT derives from a combination of the much-needed concern for patient hemodynamic stability and the habitual, center-based transfusion philosophy. However, Cave and colleagues (2019) recently demonstrated that an Hgb as low as 70 g/L (hematocrit < 21%) is a safe conservative transfusion trigger in HNC free flap surgery, with no significant increase in flap-related complications [32]. Many prominent guidelines advocate for restrictive transfusion strategies tailored to individuals [33]. Our center transfuses at the surgical team discretion—often in consult with the case anesthetist or intensivist. Acutely, the primary modality through which PBT offers lifesaving support is by maintaining normovolemia. In fact, stored RBCs are often quite depleted of 2,3-diphosphyglycerate and the left-shifted Hgb offers poor or insignificant oxygen delivery [34]. The low viscosity of the blood in the state of anemia, which enhances its oxygen delivery capabilities, is also acutely reversed with PBT. It may take up to a few days following transfusion before the cellular conditions of stored RBCs normalize to promote optimal oxygen delivery systemically [35]. Therefore, the decision to transfuse can be subjective, and surgeons must be aware of alternatives to PBT to manage decreasing Hgb levels. Transfusion medicine requires clinical reasoning and education to produce the best outcomes. Instead, the focus of care should be on prevention and possibly preoperative optimization.

Multivariable analysis demonstrated that underweight patients with low preoperative Hgb and high T stage increase the odds of requiring PBT. With the odds ratios of PBT for preoperative Hgb adjusted for BMI and T stage, although there was high variability, there was a significantly increased odds of PBT below 120 g/L Hgb. The general trend showed an increased odds of PBT with lower preoperative Hgb, although these differences may be misleading due to the above-mentioned variability in the dataset and missing data points. Furthermore, the mean preoperative Hgb was significantly lower in the transfused group. We also found an increased mean units of blood transfused for patients under 150 g/L, with a significant spike again at 120 g/L. Therefore, our estimate of magnitude corresponds with our estimate of risk below 120 g/L Hgb. Notwithstanding this, we are unable to conclude that this concentration represents a specific high-risk cut-off. Given the various findings in HNC that suggest a dose-dependency of PBT for its potential complications, a reduction in preoperative Hgb would also increase the units of blood required to stabilize the patient. Despite the intuitive relationship between anemia and transfusion, institutional and real-world practices do not consistently address anemia prior to surgery. Our findings suggest a more proactive approach for detection and prevention.

Cancer plays a role in altering enteral homeostasis and metabolic pathways [36]. Iron deficiency is also the most common micronutrient deficiency worldwide, which is often accentuated in the state of chronic disease [37]. As such, preoperative nutritional care is fundamental for patients undergoing major cancer surgery. In our HNC free flap cohort, we found that both preoperative Hgb and low BMI (as well as high T stage) were independently associated with PBT. Currently, it is unclear if increasing BMI or low perioperative albumin is beneficial to avoid postoperative complications [38,39,40]. In the case of BMI, little apart from nutritional support can be offered in the short time between the HNC diagnosis and surgery—often a matter of just a few weeks. Preoperative Hgb, however, is a key risk factor that may be modulated reliably in this time window. While a clear high-risk threshold for preoperative Hgb was not delineated, preoperative anemia as well as its severity should provoke care providers to consider Hgb optimization measures in accordance with the strong recommendations from the Enhanced Recovery After Surgery (ERAS) Society [41].

Many methods to improve preoperative anemia have been suggested over the years. Oral iron pills are quick to implement and a cheap venture to normalize iron stores. A few dollars of iron pills may mitigate a unit of blood, saving the system approximately CAD $1000 from the blood bank to administration [42]. However, patient compliance, a lower efficacy, and the time needed to alleviate preoperative anemia creates a barrier for practical usage of pre-operative oral iron supplements in major HNC surgery. Other modalities such as erythropoietin and autologous blood transfusion have also been promoted [43,44]. However, concerns of safety, added costs, and time constraints in the latter have rendered these options less favorable [45,46]. Furthermore, autologous donation can further lower Hgb and deplete iron stores. Not only is iron deficiency the most common form of anemia, there is compelling evidence in the surgical oncology literature suggesting that iron-deficiency anemia is often underdiagnosed [47,48,49,50]. Intravenous (IV) iron is a relatively cheap, safe, and effective method of priming preoperative Hgb, especially in cases where low preoperative Hgb or anemia must be addressed in a tight time window to reduce PBT and the other sequalae of preoperative anemia [51]. This therapy works almost immediately and can raise blood Hgb levels by 7.6 g/L in 2–4 weeks prior to surgery, with some centers achieving hemoglobin elevations of at least 10 g/L [52,53,54]. Given the time constraint, IV iron should be considered for the preoperative anemic patient. Including such interventions in the preoperative period could be incorporated into guidelines, such as enhanced recovery after surgery (ERAS) protocols to standardize the approach. While ERAS guidelines were implemented at the study site during the study period, there is no element that provides guidance on preoperative anemia. Intraoperative tools can also serve as a useful adjunct. Various modalities and hemostatic surgical instruments have been postulated to mitigate PBT, such as electrocautery, ultrasonic scalpels, tranexamic acid, leukofiltrated blood salvage, and methods to maintain normovolemia [30,55,56,57]. The usage of these adjunct methods (e.g., erythropoietin or tranexamic acid) was not specifically recorded at our site. Combining various innovations such as IV iron alongside erythropoietin and hemostatic agents may further enhance patient outcomes.

Apart from BMI and preoperative Hgb, other factors independently associated with PBT on multivariate analysis included high T stage (III or IV versus I or II) and the Clavien-Dindo class (IIIb–V versus I–IIIa). The former is likely a proxy for the overall magnitude of surgical resection and operation time. The latter may have some overlap with the overall complexity of surgery but also accounts for increased intraoperative blood loss due to injury to any major vessels. Both cases, however, present either largely unmodifiable or unpredictable variables in terms of short-term surgical planning.

Our quality improvement project incorporated a robust, longitudinal HNC free flap database but presents with a few limitations. Firstly, there was some missing data, and the resulting variability may influence the generalizability of the results. Intraoperative blood loss was not accounted for due to lack of availability of these data, which may confound transfusion risk. Although other reports have still found an independent influence of low preoperative Hgb or anemia on PBT outcomes, lack of information on blood loss may have influenced the relationship in our sample. Blood loss may also be increased in patients with a low BMI, but this would only further encourage correction of preoperative anemia to mitigate the effects of intraoperative blood loss. Secondly, comorbidity status was not accounted for. The necessity for transfusion is contingent on patient hemodynamic stability. In cases of comorbid conditions affecting oxygenation or volume status, such as in patients with compromised cardiovascular conditions, transfusion may need to be triggered at an earlier point—with more units of blood likely being necessitated. Also, other conditions may influence a patient’s disposition for preoperative anemia. Given that multiple variables can confound the relationship between low Hgb and the necessity for PBT, future investigations should prospectively evaluate the effect of iron transfusion for low preoperative Hgb on PBT outcomes. However, there is a proclivity for PBT to be used in difficult surgeries (i.e., higher T stage); as such, other complications of preoperative anemia must be assessed alongside PBT in major HNC surgery.

## 5. Conclusions

We found that in the major HNC surgery cohort, low preoperative Hgb in underweight patients with late-stage tumors can predispose patients to PBT. Preoperative Hgb, however, is the strongest predicator of transfusion. While there was a positive inflection in the odds and volume of PBT administered for those below 120 g/L Hgb, we were unable to confidently ascertain a singular threshold to put patients at risk of PBT. Given that preoperative anemia is a modifiable risk factor, head and neck surgeons should be encouraged to recognize at-risk patients and manage their micronutrient needs as a part of presurgical planning. Further studies should be carried out to determine the feasibility of IV iron as a method to alleviate preoperative anemia and reduce unnecessary PBT in major HNC surgery. Future work to explore the association between PBT and oncologic outcomes would be an invaluable addition to the literature.

## Figures and Tables

**Table 1:** Patient demographics and overall PBT outcome data.

Variable	*n* (%)	Transfused, *n* (%)	Non-Transfused, *n* (%)	*p*-Value
**Preoperative Variables**				
Preoperative Hgb (g/L, Mean [SD])	141.6 [16.90]	128.4 [19.96]	144.6 [14.55]	**<0.0001**
*Sex*	363	68 (19)	295 (81)	0.008 *
Male	256 (71)	39 (15)	217 (85)	
Female	107 (29)	29 (27)	78 (73)	
Age (yrs, Mean [SD])	61.4 [12.27]	62.0 [12.94]	61.3 [12.13]	0.6856 ^‡^
Comorbidity	363	68 (19)	295 (81)	0.170 **^†^**
0	149 (41)	29 (20)	120 (81)	
1	112 (31)	17 (15)	95 (85)	
2+	72 (20)	19 (26)	53 (74)	
Missing	30 (8)	3 (10)	27 (90)	
*T stage*	332	68 (19)	295 (81)	**0.007 ^†^**
T0/Tis	39 (11)	9 (23)	30 (77)	
T1	45 (12)	4 (9)	41 (91)	
T2	89 (25)	11 (12)	78 (88)	
T3	40 (11)	6 (15)	34 (85)	
T4	119 (33)	35 (29)	84 (71)	
Missing	31 (9)	3 (10)	28 (9)	
N *stage*	291	52 (18)		0.467 *
N0	149 (41)	29 (19)	120 (81)	
N+	142 (39)	23 (16)	119 (84)	
Missing	72 (20)	16 (24)	56 (19)	
*Cancer stage*	363	68 (19)	295 (81)	0.123 **^†^**
Stage 0–II	95 (26)	12 (13)	83 (87)	
Stage III–IV	238 (66)	52 (22)	186 (78)	
Missing	30 (8)	4 (6)	26 (9)	
*Smoking*	363	68 (19)	295 (81)	0.719 *
Current	111 (31)	19(17)	98 (83)	
Former	123 (34)	21 (17)	102 (83)	
Never	87 (24)	18 (21)	69 (23)	
Missing	42 (12)	10 (24)	32 (76)	
*EtOH*	363	68 (19)	195 (81)	0.546 *
Current	201 (55)	35 (17)	166 (83)	
Former	42 (12)	7 (17)	35 (12)	
Never	71 (20)	16 (24)	55 (19)	
Missing	49 (14)	10 (15)	39 (13)	
*BMI* (*categorical*)	333	65 (20)	268 (80)	**0.001** *
Underweight	24 (7)	11 (46)	13 (54)	
Normal weight	142 (43)	33 (23)	109 (41)	
Overweight	103 (31)	13 (12)	90 (34)	
Obese	64 (19)	8 (13)	56 (21)	
*BMI* (*continuous*, Mean [SD])	25.7 [5.93]	23.7 [6.04]	26.2 [5.80]	**0.0002 ^¶^**
*Cancer recurrence*	363	68 (19)	295 (81)	0.738 *
Yes	54 (15)	11 (20)	252 (82)	
No	301 (85)	57 (18)	43 (80)	
*Primary cancer site*	363	68 (19)	295 (81)	0.339 **^†^**
Oral cavity	237 (65)	41 (17)	196 (83)	
Oropharynx	16 (4)	4 (25)	12 (75)	
Hypopharynx	8 (2)	2 (25)	6 (75)	
Larynx	9 (2)	1 (11)	8 (89)	
Paranasal sinus	23 (6)	9 (39)	14 (61)	
Skin	36 (10)	4 (11)	32 (89)	
Salivary gland	14 (4)	3 (21)	11 (79)	
Thyroid	2 (1)	0 (0)	2 (100)	
Unknown	1 (0)	0 (0)	1 (100)	
Other	17 (5)	4 (24)	13 (76)	
*Type of cancer*	363	68 (19)	295 (81)	0.913 **^†^**
SCC	290 (80)	54 (19)	236 (81)	
NSCC	73 (20)	14 (19)	59 (81)	
**Intraoperative variables**				
*Type of flap*	363	68 (19)	295 (81)	**<0.0001** *
RFFF	184 (51)	17 (9)	167 (91)	
FFF	80 (22)	25 (31)	55 (69)	
ALT	55 (15)	11 (20)	44 (80)	
Other	44 (12)	15 (34)	29 (66)	
*Neck dissection*	360	67 (19)	293 (81)	0.392 *
None	67 (19)	16 (24)	51 (76)	
Right	107 (30)	15 (14)	92 (86)	
Left	89 (25)	16 (18)	73 (82)	
Bilateral	97 (27)	20 (21)	77 (79)	
*Clavien-Dindo class*	332	65 (20)	267 (80)	**<0.001 ^†^**
0–IIIa	280 (84)	46 (16)	234 (84)	
IIIb–V	52 (16)	19 (37)	33 (63)	

* Chi-square test; **^†^** Fisher’s exact test; ^‡^ Student’s *t*-test; ^¶^ Wilcoxon rank-sum test. Abbreviations: SD = standard deviation, Hgb = hemoglobin, T stage = tumor stage, N stage = node stage, EtOH = alcohol, BMI = body mass index, SCC = squamous cell carcinoma, NSCC = non-squamous cell carcinoma, RFFF = radial forearm free flap, FFF = free fibular flap, ALT = anterolateral thigh flap.

**Table 2:** Multivariate logistic regression for predictors of PBT.

Predictor	*p*-Value	Odds Ratio
Preoperative Hgb	<0.0001	0.94
Sex	ns	0.90
Cancer Stage	0.049	2.07
BMI	0.040	5.53
Clavien-Dindo Class	0.007	3.23

Abbreviations: Hgb = hemoglobin, BMI = body mass index.

## Data Availability

The datasets generated and/or analyzed during the current study are available from the corresponding author on reasonable request.

## Supplementary Materials

The following supporting information can be downloaded at https://www.mdpi.com/article/10.3390/cancers17132136/s1, Table S1. STROBE checklist.
